# *Ocimum sanctum* Linn. Extract Improves Cognitive Deficits in Olfactory Bulbectomized Mice via the Enhancement of Central Cholinergic Systems and VEGF Expression

**DOI:** 10.1155/2021/6627648

**Published:** 2021-06-30

**Authors:** Xoan Thi Le, Hien Thu Nguyen, Tai Van Nguyen, Hang Thi Nguyet Pham, Phuong Thi Nguyen, Khoi Minh Nguyen, Ba Van Nguyen, Kinzo Matsumoto

**Affiliations:** ^1^Department of Pharmacology and Biochemistry, National Institute of Medicinal Materials, Hanoi 10000, Vietnam; ^2^Department of Phytochemistry, National Institute of Medicinal Materials, Hanoi 10000, Vietnam; ^3^Oncology Center of 103 Military Hospital, Vietnam Military Medical University, Hanoi 10000, Vietnam; ^4^Center for Supporting Pharmaceutical Education, Daiichi University of Pharmacy, Fukuoka 815-8511, Japan; ^5^Graduate School of Pharmaceutical Sciences, Daiichi University of Pharmacy, Fukuoka 815-8511, Japan

## Abstract

This study aimed to clarify the antidementia effects of ethanolic extract of *Ocimum sanctum* Linn. (OS) and its underlying mechanisms using olfactory bulbectomized (OBX) mice. OBX mice were treated daily with OS or a reference drug, donepezil (DNP). Spatial and nonspatial working memory performance was measured using a modified Y maze test and a novel object recognition test, respectively. Brain tissues of the animals were subjected to histochemical and neurochemical analysis. OS treatment attenuated OBX-induced impairment of spatial and nonspatial working memories. OBX induced degeneration of septal cholinergic neurons, enlargement of the lateral ventricles, and suppression of hippocampal neurogenesis. OS and DNP treatment also depressed these histological damages. OS administration reduced *ex vivo* activity of acetylcholinesterase in the brain. OBX diminished the expression levels of genes coding vascular endothelial growth factor (VEGF) and VEGF receptor type 2 (VEGFR2). Treatment with OS and DNP reversed OBX-induced decrease in VEGF gene and protein expression levels without affecting the expression of the VEGFR2 gene. These results demonstrate that the administration of OS can lessen the cognitive deficits and neurohistological damages of OBX and that these actions are, at least in part, mediated by the enhancement of central cholinergic systems and VEGF expression.

## 1. Introduction

Alzheimer's disease (AD) is a progressive neurodegenerative disorder characterized by impairment of cognitive functions including memory, language, attention, comprehension, reasoning, and judgment. AD mainly affects elderly people and is the most common type of dementia. The development of new drugs or strategies which are effective for prevention/therapy of dementia and safe for long-term use is needed. Many studies have been conducted to explore traditional medicines or medicinal plants with few reported side effects and are readily available for long-term therapy.

*Ocimum sanctum* Linn. (OS), also known as *Ocimum tenuiflorum*, is an aromatic plant in the family Lamiaceae, a widely distributed and cultivated plant grown throughout the Southeast Asian tropics. Leaves of this plant contain essential oils, flavonoids (apigenin, luteolin, apigenin-7-O-glucuronide, orientin, and olludistin), triterpenoids (oleanolic and ursolic acid), polyphenols, and tannins [1–4]. This plant has long been used as tea herbs/a cuisine material in Vietnam and other Southeast Asian countries. Besides, OS is recognized for its various therapeutic properties. Indeed, in Ayurvedic medicine, OS has been used as an adaptogen to help adapt to stress by balancing different body physiological processes. Thus, daily intake of this herb has been believed to improve physical and mental health and promote physiological and cognitive function, thereby providing longevity [5–8].

These clinical applications of OS have been supported by various biological and pharmacological studies exposing its anticarcinogenic [9], antidiabetic [10], anti-inflammatory [11], and antistress activities [3, 12]. Recent evidence suggests that this plant's aqueous extracts exhibit antidementia properties in animal models of cognitive deficits, such as transient cerebral ischemia [13] and scopolamine-, diazepam-, and aging-induced amnesia [14]. Inhibiting acetylcholinesterase activity has been suggested as a possible mechanism of the antidementia action of OS constituents [15]; however, the exact mechanism of action of OS remains unclear.

To further investigate the potential therapeutic effects of OS for AD and its underlying mechanisms, this study used mice with surgical removal of their olfactory bulb. Olfactory bulbectomized (OBX) rodent models have been widely used to investigate the effects of drugs on emotional deficits such as endogenous depression, as well as cognitive dysfunctions like AD [16–20]. Evidence shows that OBX causes various behavioral symptoms similar to AD [21, 22] and induces increased levels of amyloid precursor protein and amyloid-*β* [21, 23]. It is also thought to lead to the dysfunction of various neurotransmitter systems in the brain, including the serotonergic, cholinergic, and glutamatergic systems [17, 18, 24, 25]. These neurobiological and pathophysiological features of OBX offer a useful animal model to evaluate new drug therapies, including medicinal plants for the prevention/treatment of AD [17, 18, 20].

This study investigates the antidementia effects of OS extract and the mechanisms underlying its action using the OBX model of mice. We focused on the cholinergic and VEGF systems for a couple of reasons. First, previous studies have demonstrated that a close linkage exists between cholinergic systems and VEGF signaling systems in the brain and contributes to the neuroprotective and antidementia effects of tacrine, an acetylcholinesterase inhibitor [26, 27]. Second, it has been reported that elevated cerebrospinal fluid VEGF are associated with slower rates of hippocampal atrophy and slower rates of cognitive decline, particularly among individuals with elevated levels of AD biomarkers on human [28]. The present results illustrated the effect of OS on OBX-induced cognitive deficits and neurodegenerative symptoms. These OS effects were at least in part mediated by enhancement of central cholinergic systems and normalization of VEGF expression.

## 2. Materials and Methods

### 2.1. Animals

Male *Swiss albino* mice (7-8 weeks old) were supplied by the National Institute of Hygiene and Epidemiology, Hanoi, Vietnam. The mice were habituated to the laboratory animal room for at least one week before starting the experiments. The animal room was maintained at 25 ± 1°C with 65–75% humidity and a 12-hour light/dark cycle (lights on from 7:00 to 19:00). Commercial laboratory food and tap water were provided *ad libitum*. Animals were subjected to behavioral experiments during the light phase from 9:00 to 18:00. All studies have been performed in accordance with the Guide for the Care and Use of Animals (NIH publication #85–23, revised in 1985) and were approved by the Institutional Animal Use and Care Committees of the National Institute of Medicinal Materials (NIMM), Hanoi, Vietnam.

### 2.2. Preparation and Chemical Profiling of Plant Extract

*Ocimum sanctum* (OS) used in this study was collected in Hanoi city, Vietnam, in 2016 and identified by Dr. Pham Thanh Huyen (Department of Medicinal Plant Resources, NIMM, Vietnam). A voucher specimen of the herb was deposited in NIMM (voucher specimen #: NIMM-16474B). In this study, the 70% ethanol extract of OS was prepared and used for the experiments for several reasons. First, a phytochemical feature of OS is to contain essential oils, flavonoids, terpenoids, polyphenols, and tannins [1–4], which are efficiently extracted by 70% EtOH [1]. Second, in the case that the antidementia effects of OS and their underlying mechanisms are demonstrated by using 70% EtOH extract of OS, we can expect to efficiently isolate and identify the chemical constituents contributing to the effects of OS *in vivo* by further studies. For plant extract preparation, the aerial part of OS (356 g) was cut into small pieces, dried in a hot air oven at 50°C, and then crushed. The powdered herb was extracted with 70% ethanol at the ratio of 1 : 7 (*w*/*v*) for 2 hours in a reflux system. This reflux extraction was repeated three times, and the samples were filtered. They were then combined and concentrated under reduced pressure at 50°C. The extract was dried in a vacuum oven at 50°C and kept at 4°C until use. The extraction yield from the starting dried herb was calculated as 16.5% (*w*/*w*).

Chemical profiling of OS extract and quantification of some chemical constituents were performed using high-performance liquid chromatography with a UV detector (HPLC-UV) equipped with a 5 *µ*m Vertisep® C18 column. The Materials and Methods of HPLC analysis were shown in detail in the supplemental data. The analysis revealed that the OS extract contained 1.02% ursolic acid, 1.48% oleanolic acid, 0.10% apigenin, 0.11% luteolin, 3.06% luteolin-7-O-glucuronide, and 0.85% apigenin-7-O-glucuronide.

### 2.3. Surgical Operation and Drug Administration

Surgical operation of OBX in mice was conducted as previously described [17, 18] on day 0. The mice were anesthetized by injecting sodium pentobarbital at a dose of 60 mg/kg (i.p.). The mice were placed in a stereotactic instrument (Narishige, Tokyo, Japan). The skull covering the olfactory bulbs was exposed by a skin incision under local anesthesia (1% lidocaine). A 1 mm burr hole was made at the skull covering the bulbs using a surgical drill. The bulbs were removed from the mouse brain by aspiration through a syringe. Then a hemostatic gelatin sponge was placed in the gap where the bulbs had been. Sham operation was conducted similarly without removing the bulb.

**Figure 1:**
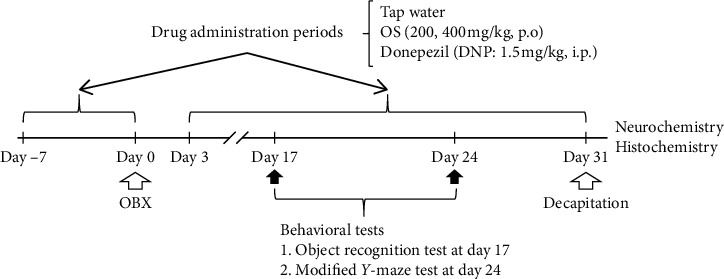
Schematic drawing of the experimental protocol. *Swiss albino* mice were treated daily with *Ocimum sanctum* extract (OS; 200, 400 mg/kg, p.o.), donepezil (DNP; 1.5 mg/kg, i.p.), or tap water. Object recognition test and the modified Y maze tests were performed on day 17 and day 24, respectively. After completing these behavioral tests, the animals were sacrificed, and the brain tissues were collected for histochemical and neurochemical studies.

The OS extract was suspended in distilled water and administered daily at doses of 200 and 400 mg/kg (p.o.). Donepezil HCl (DNP; Tokyo Chemical Industry Co., Ltd, Japan), a reference drug, was reconstituted in 0.9% saline and administered daily at a dose of 1.5 mg/kg (i.p.). Sham and OBX vehicle-treated control groups were administered tap water orally (p.o.). Mice in the drug treatment groups received drug administration 1 week (day −7) before surgery, and these treatments were resumed from day three after the surgery. On the day of the OBX surgery and behavioral tests, drugs were administered one hour before the test. This procedure is summarized in Figure 1.

### 2.4. Behavioral Tests

#### 2.4.1. Novel Object Recognition Test (ORT)

**Figure 2:**
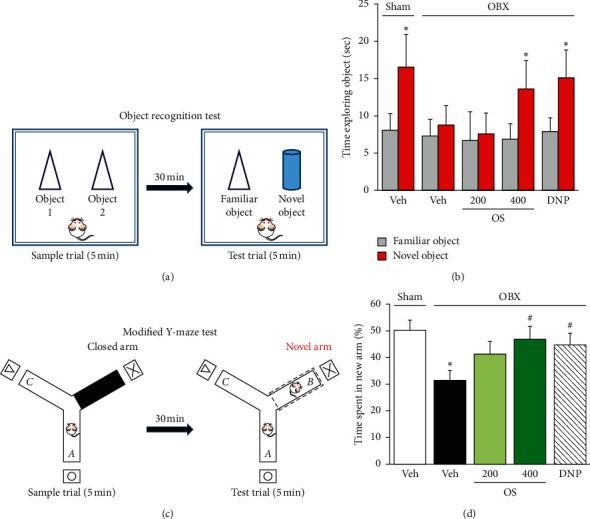
OS and DNP attenuated OBX-induced impairments of working memory in mice. (a) Experimental protocol of the object recognition test (ORT). (b) Nonspatial working memory performance of sham and OBX groups in ORT. ^*∗*^*p* < 0.05 comparing time exploring novel versus familiar objects. (c) Experimental protocol of the modified Y-maze test. (d) Spatial short-term working memory performance of sham and OBX groups in modified Y-maze test. Data are expressed as % time animals spent exploring the novel arm in the test session (mean ± SEM) (*n* = 9–12). ^*∗*^*p* < 0.05 comparing OBX to sham groups, ^#^*p* < 0.05 versus vehicle-treated OBX group.

The ORT relies on rodents' natural proclivity for exploring novelty and is used to test recognition memory. According to our previous study [17, 18, 29], this test was carried out with minor modification on day 17 after OBX (Figure 1). Briefly, 24 hours before the test, mice were individually placed in an examination box (35 × 35 × 50 cm) for 10 minutes. The ORT consisted of two sessions, a 5 min familiarization and a 5 min test that were separated with a 30 min interval. During the familiarization session, two identical objects, object 1 and object 2, were placed separately in the observation box and each mouse was allowed to explore the arena freely for five minutes (Figure 2(a)). During the test session, one of the previously explored objects was replaced with a novel object. The experimental apparatus, including the objects, was cleaned with 70% ethanol to remove odor cues between sessions. The animals' behavior was video-recorded, and the time spent exploring each of the two objects was analyzed using ANY-maze software (ver. 4.99, Stoelting Co., IL, USA). Exploration time was defined by animal nose-point detection within a 2 cm radius around the object.

#### 2.4.2. Modified Y-Maze Test

One week after the ORT test, mice were subjected to the modified Y-maze test, conducted as previously described [17, 18, 30]. The Y-maze apparatus consists of black polypropylene walls with three arms; each arm had a length of 40 cm, a height of 18 cm, a width of 12 cm at the top, and 3 cm at the bottom. Different spatial cues surrounded the maze. The test phase was conducted 30 minutes after the familiarization phase. During the familiarization session, each mouse was individually placed in the maze, where one of the three arms was closed (Figure 2(c)). The mouse was allowed to explore the two arms freely for five minutes. During the test session, all three arms were opened, and the previously closed arm was defined as the novel arm. The animal returned to the maze and allowed to explore the arms freely for five minutes. The entire experimental apparatus was cleaned with 70% ethanol to remove odor cues between sessions. Animal behavior was video-recorded and analyzed using ANY-maze software ver. 4.99 (Stoelting).

### 2.5. Histological Study

Animals were anesthetized with sodium pentobarbital (60 mg/kg, i.p.), and the tissues were fixed by intracardiac perfusion with 4% paraformaldehyde (PFA) in phosphate-buffered saline (PBS). After perfusion, the brains were collected and postfixed with 4% PFA/PBS for 24 hours at 4°C and then embedded in paraffin. Serial coronal brain sections (5 *μ*m) were cut on a microtome (Microm HM 340 E, Thermo Fisher Scientific, Dreieich, Germany) and stored at 4°C.

#### 2.5.1. Microscopic Analysis of Lateral Ventricles

The brain sections at ≈ −1.46 mm from the Bregma were stained with cresyl violet and used to calculate the size of the lateral ventricle. Lateral ventricles were observed on an Olympus PROVIS® microscope (Olympus Inc., Tokyo, Japan) under ×1.25 object magnification. The size of the area of the lateral ventricles was calculated using Image J software (ver. 1.41; NIH, MD, USA), and changes in the size of the lateral ventricle area were calculated as the ratio of the total area of the lateral ventricles to the total brain area.

#### 2.5.2. Immunohistochemistry

Cholinergic neurons in the medial septum and newly generated neurons in the hippocampal dentate gyrus were analyzed by staining choline acetyltransferase- (ChAT-) immunopositive cells and doublecortin- (DCX-) immunopositive cells, respectively. The paraffin-embedded sections were deparaffinized. After washing with PBS, the sections were treated with Target Retrieval Solution (Dako Japan Inc., Tokyo, Japan) and incubated at 95°C in a water bath for 30 minutes. The brain tissues were then incubated in 0.1% hydrogen peroxide for 30 minutes and blocked with 5% bovine serum albumin in PBS for 1 hour at room temperature. ChAT goat polyclonal antibody (1 : 200 dilution, AB-144P; Millipore, CA, USA) or DCX rabbit polyclonal antibody (1 : 200 dilution, ab18723, Abcam, Cambridge, UK) was applied to the brain sections for 24 hours at 4°C. After washing with PBS, the sections were incubated in secondary antibodies conjugated to horseradish peroxidase: bovine anti-goat IgG antibody (1 : 100 dilution, sc-2350, Santa Cruz Biotechnology Inc., Dallas, TX), or anti-rabbit IgG antibody (1 : 200 dilution, #4074, Cell Signaling Tech., MA, USA) for one hour. A reaction product was detected using a 3,3′-diaminobenzidine tetrahydrochloride (DAB), and the brain sections were then counterstained with hematoxylin solution. Sections were analyzed using the Olympus PROVIS® microscope under ×10 object magnification. ChAT-positive cells in the medial septum area (200 × 100 *μ*m^2^) of brain sections at bregma ≈ +0.98 mm and DCX-positive cells in the dentate gyrus area of brain sections at bregma ≈ −1.70 mm (2-3 sections of each mouse) were counted in a blind manner.

### 2.6. Neurochemical Studies

For neurochemical studies, animals were sacrificed under pentobarbital anesthesia, and the brain was removed. Hippocampi and frontal cortices were quickly separated from the mouse brain and frozen in liquid nitrogen. The brain tissues were stored at −80°C until use.

#### 2.6.1. Quantitative Real-Time Polymerase Chain Reaction (qRT-PCR)

The expression levels of vascular endothelial growth factor (VEGF) and VEGF receptor 2 (VEGFR2) genes in the hippocampus of mice were analyzed using quantitative real-time PCR (qRT-PCR). qRT-PCR was conducted as previously described [17, 31, 32]. Total RNA was extracted from the hippocampus using Sepazol® (Nacalai Tesque, Kyoto, Japan). Oligo (dT) primers and M-MLV reverse transcriptase® (Invitrogen, Tokyo, Japan) were used to synthesize the cDNA. QRT-PCR was performed in the Step One Real-time PCR System® (Applied Biosystems, Waltham, MA, USA) using the cDNA as a template and Fast SYBR Green Master Mix (Applied Biosystems, Waltham, MA, USA). The primer sets for VEGF, VEGFR2, and *β*-actin are shown in Table 1. Melting curve analysis of each gene was performed after every amplification cycle. Standard curves of genes (*R* > 0.99) were generated by plotting the log concentration of each gene versus cycle threshold. In all reactions, *β*-actin mRNA was used to normalize the results of the target genes.

#### 2.6.2. Western Blot Analysis

Western blotting was performed by slightly modifying the method described previously [26, 32]. Briefly, the hippocampus was homogenized in protein lysis buffer. Total protein (20 *µ*g) prepared from each sample was separated on a 12% SDS-PAGE gel and transferred to PVDF membranes (Bio-Rad Laboratory, CA, USA). The membranes were blocked in a 5% nonfat milk for 1 h at RT and then probed with VEGF rabbit polyclonal antibody (A-20: sc-152, 1 : 1000 dilution, Santa Cruz Biotechnology) and *β*-actin rabbit polyclonal antibody (PA1-183, 1 : 2000 dilution, Thermo scientific) at 4°C overnight. Membranes were washed 3 times in TBS-T and then incubated with anti-rabbit secondary antibody linked with horseradish peroxidase (Cell Signaling) for 1 hr at RT. The blots were developed using Amersham™ ECL™ Prime (GE Healthcare, Buckinghamshire, UK). A flatbed scanner was used to scan the photos taken on autoradiography film (Santa Cruz Biotechnology) for image digitalization. Then, the densitometric analysis was conducted using Image J software ver. 1.41. (NIH).

#### 2.6.3. *Ex Vivo* Measurements of Acetylcholinesterase (AChE) Activity

Determination of AChE activity in the mouse brain was performed using the colorimetric method as previously described with slight modification [17, 30, 33]. Briefly, the frozen frontal cortex was homogenized in 20 times its volume of PBS (pH 7.4) containing 1% Triton X-100. After centrifugation (15,000 ×g, 4°C, 20 minutes), the supernatant was collected. The enzyme reaction was performed in a 96-well microplate by adding 10 *μ*l aliquots of the supernatant, 20 *μ*l of 10 mM Ellman's Reagent (Sigma-Aldrich, MO, USA), 20 *μ*l of 30 mM acetylthiocholine iodide (Sigma-Aldrich), and 160 *μ*l of PBS to each well. The spectrophotometric absorption at 405 nm was measured in a microtiter plate reader (HumaReader HS; Human Diagnostics, Wiesbaden, Germany) during a five-minute incubation period at 25°C. The enzyme activity was expressed as nmol AChE hydrolyzed/mg tissue/min.

### 2.7. Data Analysis

SigmaStat® software (ver. 3.5, SYSTAT Software Inc., Richmond, CA, USA) was used for statistical analyses. The data were presented as the mean ± SEM. All data except ORT were analyzed by one-way analysis of variance (ANOVA) followed by *post hoc* Student–Newman–Keuls test. Paired Student's *t*-test was used for statistical analysis of the ORT data. Values of *p* < 0.05 were considered significant.

## 3. Results

### 3.1. OS as well as DNP Improves Working Memory Deficits Caused by OBX

The ORT was employed to elucidate the effects of OS extract on nonspatial short-term working memory. As shown in Figures 2(a) and 2(b), the sham group and DNP-treated OBX group significantly spent more time exploring the new object than the familiar one (sham group: *t* = −3.320, df = 8, *p*=0.011; DNP-treated OBX group: *t* = −2.842, df = 11, *p*=0.016), whereas the vehicle-treated OBX group exhibited no preference for exploring the novel object (*t* = −1.109, df = 11, and *p*=0.291). These results suggest that OBX causes impairment of nonspatial working memory in a manner which can be reversed by the daily administration of DNP. Similarly, the daily administration of OS (200–400 mg/kg) dose-dependently increased the time OBX animals spent exploring the novel object (OS 200 mg/kg—treated OBX group: *t* = −0.695, df = 8, *p*=0.507; OS 400 mg/kg—treated OBX group: *t* = −3.092, df = 9, *p*=0.013).

We also examined the effect of OS on the spatial working memory performance of OBX animals using the modified Y-maze test. As shown in Figures 2(c) and 2(d), time spent in the novel arm by the vehicle-treated OBX group was significantly shorter than that of the sham group, indicating an impairment of spatial working memory. The administration of OS (200–400 mg/kg) dose-dependently attenuated OBX-induced deficits in spatial working memory. Again, DNP treatment (1.5 mg/kg) significantly ameliorated the impairment of spatial working memory of OBX mice (*F* (4, 46) = 3.530, *p*=0.014; vehicle-treated OBX group vs. sham group, *p*=0.013; vehicle-treated OBX group vs. OS 200 mg/kg-treated OBX group, *p*=0.085; vehicle-treated OBX group vs. OS 400 mg/kg-treated OBX group, *p*=0.038; vehicle-treated OBX group vs. DNP-treated OBX group, *p*=0.034).

### 3.2. OS Reduces OBX-Induced Neurodegeneration of ChAT-Immunopositive Cells in the Medial Septum

**Figure 3:**
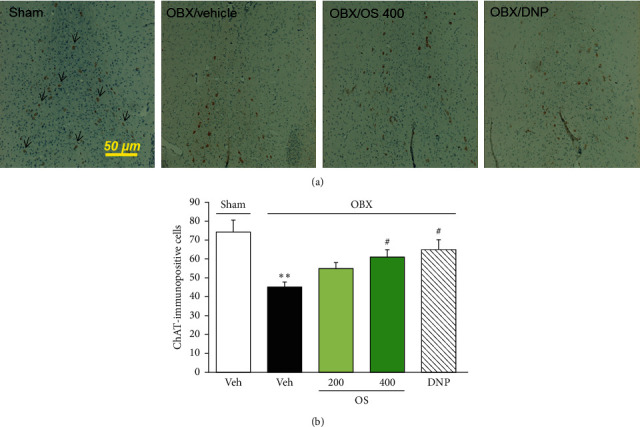
OS and DNP reversed OBX-induced neurodegeneration of cholinergic neurons in the medial septum (MS). (a) Typical images of choline acetyltransferase- (ChAT-) positive cells in the MS. The brain sections (2-3 sections/mouse) at bregma  ≈ +0.98 were stained with ChAT antibody. Scale bar = 50 *μ*m. Arrows indicate ChAT-positive cells. (b) Quantitative analyses of the number of ChAT-positive cells in the MS. Values are represented as mean ± SEM (*n* = 3-4). ^*∗∗*^*p* < 0.01 versus sham group; ^#^*p* < 0.05 versus vehicle-treated OBX group.

Consistent with previous reports [17, 25], OBX significantly reduced the number of ChAT-immunopositive cells in the medial septum in a manner which can be prevented by the acetylcholinesterase inhibitor DNP, implying degeneration of cholinergic neurons (Figure 3). Treatment of OBX animals with OS (200–400 mg/kg/day) dose-dependently alleviated the OBX-induced degeneration of medial septum cholinergic neurons (*F* (4, 12) = 7.678, *p*=0.003; vehicle-treated OBX group vs. sham group, *p*=0.002; vehicle-treated OBX group vs. OS 200 mg/kg-treated OBX group, *p*=0.103; vehicle-treated OBX group vs. OS 400 mg/kg-treated OBX group, *p*=0.022; vehicle-treated OBX group vs. DNP-treated OBX group, *p*=0.017).

### 3.3. OS Prevents Enlargement of Lateral Ventricles and Suppression of Hippocampal Neurogenesis Caused by OBX

**Figure 4:**
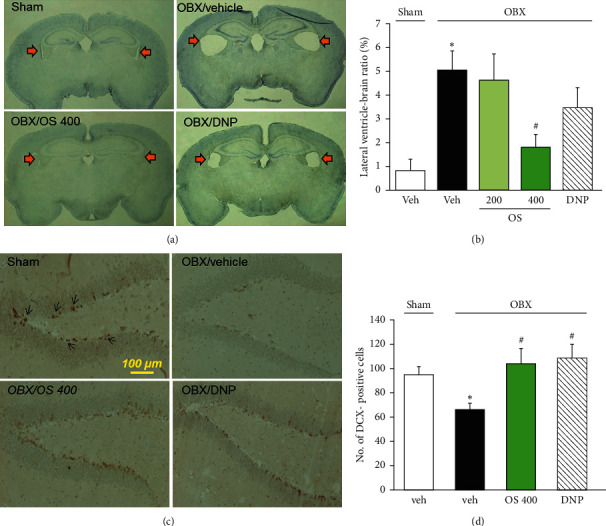
Effects of OS and DNP on the enlargement of lateral ventricle and the suppression of hippocampal neurogenesis caused by OBX. (a) Typical images of brain sections located at ≈ −1.46 mm relative to the bregma. Arrows indicate lateral ventricles. (b) Quantitative analyses of lateral ventricle area in the brain sections. The lateral ventricle-brain ratio index was calculated as the ratio of total both lateral ventricles area to total brain area. (c) Typical photos of DCX-positive cells in the dentate gyrus of the hippocampus. Scale bar = 100 *μ*m, bregma ≈ −1.70 mm. Arrows indicate DCX-positive cells. (d) Quantitative comparisons of the number of DCX-positive cells in the hippocampus among the different groups. Each data column represents the mean ± SEM (*n* = 3–5). ^*∗*^*p* < 0.05 versus sham group and ^#^*p* < 0.05 versus vehicle-treated OBX group.

As shown in Figures 4(a) and 4(b), the vehicle-treated OBX mice had significantly enlarged lateral ventricles compared to the sham-operated animals (*F* (4, 13) = 6.7, *p*=0.004; vehicle-treated OBX group versus sham group, *p*=0.006). This histological changes caused by OBX were dose-dependently mitigated in the OBX groups treated with OS (200–400 mg/kg/day) (vehicle-treated OBX group versus OS 200 mg/kg-treated OBX group, *p*=0.693; vehicle-treated OBX group versus OS 400 mg/kg-treated OBX group, *p*=0.023). In contrast, the administration of DNP tended to suppress the OBX-induced enlargement of the lateral ventricles, but the effect was insignificant (*p*=0.693).

These results, paired with the position of the lateral ventricles as a brain region where neurogenesis actively occurs to supply neurons to the olfactory bulb even in adults, led us to examine whether neurogenesis in the hippocampal dentate gyrus is susceptible to OBX and OS treatment. As shown in Figures 4(c) and 4(d), the number of cells immunopositive to DCX, a marker of young neurons, in the dentate gyrus region was significantly reduced in the vehicle-treated OBX group than the sham-operated group (*F* (3, 12) = 4.568, *p*=0.023; vehicle-treated OBX group vs. sham group, *p*=0.044). Moreover, the daily administration of OS (400 mg/kg) and DNP (1.5 mg/kg) completely abolished OBX-induced suppression of neurogenesis in the hippocampal dentate gyrus (vehicle-treated OBX group vs. OS 400 mg/kg-treated OBX group, *p*=0.019; vehicle-treated OBX group vs. DNP-treated OBX group, *p*=0.035).

### 3.4. Effect of OS Treatment on the VEGF System in the Hippocampus

**Figure 5:**
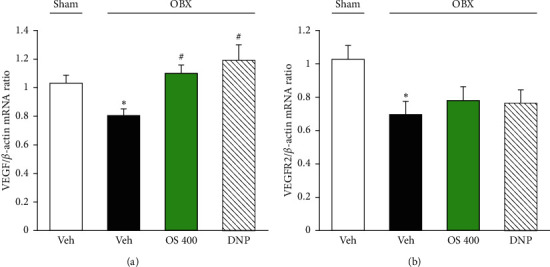
Effects of OS and DNP treatment on the expression levels of VEGF gene (a) and VEGFR2 gene (b) in the hippocampi of OBX mice using real-time PCR. *β*-Actin mRNA was used to normalize the quantities of VEGF and VEGFR2 genes. Each data column represents the mean ± SEM (*n* = 6-7). ^*∗*^*p* < 0.05 versus sham group and ^#^*p* < 0.05 versus vehicle-treated OBX group.

Real-time PCR was employed to examine the effect of OS treatment on VEGF and VEGFR2 gene expression.  Figure 5 displays the markedly decreased expression levels of VEGF and VEGFR2 in the hippocampus of vehicle-treated OBX mice compared to vehicle-treated sham group (VEGF: *F* (3, 17) = 4.346, *p*=0.049; vehicle-treated OBX group vs. sham group, *p*=0.047; VEGFR2: *F* (3, 17) = 6.530, *p*=0.004; vehicle-treated OBX group vs. sham group, *p*=0.029). OS (400 mg/kg) and DNP treatments reversed the OBX-induced reduction of VEGF gene expression (vehicle-treated OBX group versus OS-treated OBX group, *p*=0.033; vehicle-treated OBX group versus DNP-treated OBX group, *p*=0.018). However, OS and DNP treatments failed to change the expression of VEGFR2 (vehicle-treated OBX group versus OS-treated OBX group, *p*=0.239; vehicle-treated OBX group versus DNP-treated OBX group, *p*=0.252).

**Figure 6:**
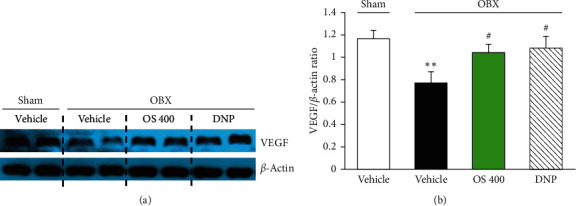
Effects of OS and DNP treatment on VEGF expression level in the hippocampi of OBX mice. (a) Representative photographs of western blotting data analysis. (b) Quantitative comparisons of the expression level of VEGF. Each data column represents the mean ± SEM (*n* = 5). Values are mean ± SEM. ^*∗∗*^*p* < 0.01 versus sham group; ^*∗*^*p* < 0.05 versus vehicle-treated OBX group.

The expression of VEGF protein was further examined by western blot. Consistent with the quantitative PCR data, the vehicle-treated OBX group had significantly reduced VEGF protein expression in the hippocampus compared with the sham-operated group (*F* (3, 16) = 5.467, *p*=0.009; vehicle-treated OBX group versus sham group, *p*=0.007). OBX-induced downregulation of VEGF expression was reversed by OS (400 mg/kg) treatment as well as DNP treatment (vehicle-treated OBX group versus OS 400-treated OBX group, *p*=0.019; vehicle-treated OBX group versus DNP-treated OBX group, *p*=0.022) (Figure 6).

### 3.5. Effects of OS on the Cholinergic System in the Brain

**Figure 7:**
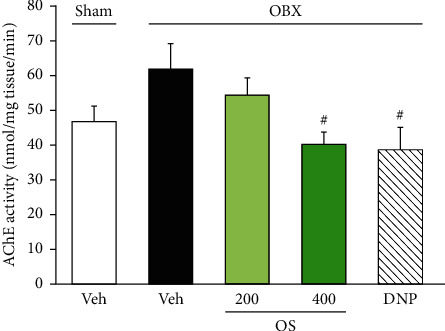
Effects of OS and DNP treatment on the *ex vivo* acetylcholinesterase (AChE) activity in the frontal cortices of OBX mice. Each data column represents the mean ± SEM (*n* = 5–7). ^#^*p* < 0.05 versus vehicle-treated OBX group.

To examine the possible involvement of endogenous acetylcholine in the action of OS, we studied the *ex vivo* activities of AChE in the frontal cortices of mice. As shown in Figure 7, cortical AChE activity of the sham and vehicle-treated OBX groups was not significantly different (*F* (4, 26) = 3.167, *p*=0.03; vehicle-treated OBX group versus sham group, *p*=0.152). However, the activities of cortical AChE in the OBX groups treated with OS (400 mg/kg) and DNP were significantly lower than that of the vehicle-treated OBX mice (vehicle-treated OBX group versus OS-treated OBX group, *p*=0.037; vehicle-treated OBX group versus DNP-treated OBX group, *p*=0.046).

## 4. Discussion

In this study, we investigated the effects of OS extract on cognitive deficits in a mouse model of dementia. The present results illustrate the ameliorative effects of OS on OBX-induced cognitive deficits in mice. The results also suggest that these effects are at least in part mediated by hippocampal neurogenesis via stimulation/protection of central cholinergic systems and restoration of VEGF expression.

We first examined the effects of OS on spatial and nonspatial short-term working memories in OBX animals using the modified Y-maze test and novel object recognition test, respectively, since working memory is reportedly impaired at an early stage of AD patients [34]. The results revealed that OBX animals exhibited significantly impaired working memory performance in both tests in a manner that can be reversed by administrating DNP, an acetylcholinesterase inhibitor, and OS extract. The remedial effects of DNP observed in this study are consistent with previous reports [17, 18, 20, 25], supporting the theory that dysfunction of central cholinergic systems is involved in the spatial and nonspatial working memory deficits of OBX mice. Given the susceptibility of OBX-induced cognitive deficits to DNP, the present results raise the possibility that OS could have a similar mechanism.

Interestingly, the present histological study revealed that when administered systemically, OS extract (400 mg/kg) but not DNP prevented the enlargement of the lateral ventricles caused by OBX in mice. The OBX-induced enlargement of the ventricles observed in the present study using *Swiss albino* mice is consistent with our previous study using ddY mice [17]. Evidence indicates that hemispheric atrophy due to ventricular enlargement is correlated with cognitive dysfunction [35] and increased incidences of senile plaques and neurofibrillary tangles in AD patients [36]. Moreover, it is reported that the dilatation of cerebral ventricles is a short-term marker for symptom progression in patients with mild cognitive impairment and AD [37]. Combined with the existing literature, the results of this study provide strong support for the use of OBX mice as an animal model of AD and suggest that OS possesses a protective effect on brain atrophy, a neurodegenerative symptom related to AD.

Another important finding in this study is that the daily administration of OS extract and DNP attenuated OBX-induced decrease in hippocampal neurogenesis. Neurogenesis in the subgranular zone of the hippocampal dentate gyrus is actively occurring and playing a significant role in the process of learning and memory formation [18, 38–40]. In fact, dysregulation of hippocampal neurogenesis is implicated in cognitive deficits in AD patients and various AD models, including OBX animals [38, 41–43]. Furthermore, evidence indicates that the septohippocampal cholinergic pathway activity plays a crucial role in the regulation of neurogenesis, even in the adult hippocampus, including proliferation, differentiation, and survival of neural stem cells [44–47]. Consistent with these findings, in the present study, OBX animals exhibited a significantly reduced prevalence of DCX-positive cells, a marker of immature neurons in the hippocampus in a manner that was reversed by DNP [46, 47]. Considering these findings, it is likely that at least two mechanisms mediate the suppressive effect of OS extract on the OBX-induced decrease in DCX-positive immature hippocampal neurons. First, OS enhanced the central cholinergic function by acting like DNP, thereby eliminating the downregulation of the hippocampal neurogenesis caused by OBX. This idea seems to be supported by the present results that the daily administration of OS extract and DNP inhibited the *ex vivo* activity of AChE in the brain tissue. Second, OS extract may contain chemicals that can enhance neurogenesis by directly acting on the hippocampal neural stem cells. Further investigations are needed to clarify the exact mechanism underlying the OS extract's effects on the hippocampal neurogenesis in OBX animals.

Additionally, the administration of OS extract and DNP normalized the downregulated expression of VEGF in the hippocampus of OBX animals. VEGF is an important signaling molecule that induces proliferation, migration, and resistance to apoptosis of endothelial cells. In the CNS, VEGF and its receptors (including VEGFR2) are ubiquitously expressed. They are mainly in choroid plexus epithelial cells, astrocytes, neurons, and neural progenitor cells [26, 48]. Moreover, VEGF expression reportedly improves cognitive deficits via promoting neurogenesis and protecting endothelial cells and neurons during ischemic conditions [49, 50]. Thus, the downregulated expression of hippocampal VEGF in OBX animals allows us to speculate that OBX leads to the reduction of hippocampal neurogenesis and the cognitive deficits are, at least in part, via dysfunction in the VEGF system in the brain. Given VEGF's neurophysiological roles, the VEGF system is likely involved in the mechanism(s) by which OS and DNP improve cognitive deficits and enhance hippocampal neurogenesis. This idea can be supported by the data from the present and previous studies. In this study, we found that the administration of OS extract inhibited AChE activity in the brain at the dose which attenuated cognitive dysfunction and the downregulated expression of VEGF in the OBX animals. Previous studies using the hippocampal slice culture demonstrated that tacrine, an AChE inhibitor, exhibited neuroprotective/neurorescuing effects in part via endogenous acetylcholine- (Ach-) mediated enhancement of VEGF expression and VEGFR2-mediated signaling [26]. Moreover, Kimura et al. [27] found that endogenous ACh plays an upregulatory role for VEGF expression in neurons and astrocytes via different mechanisms and that endogenous ACh-induced increases in VEGF levels appear to activate VEGFR2 on cholinergic neurons in the medial septal area, a nuclear origin of cholinergic neurons mainly projecting to the hippocampus. Thus, it is likely that OS and DNP enhanced the expression levels of the VEGF gene and its protein via enhancing the cholinergic systems.

The present chemical analysis revealed that the OS extract contained 1.02% ursolic acid, 1.48% oleanolic acid, 0.10% apigenin, 0.11% luteolin, 3.06% luteolin-7-O-glucuronide, and 0.85% apigenin-7-O-glucuronide. However, it is unclear which chemical component(s) can account for the ameliorative effects of OS extract on OBX-induced cognitive deficits. Wang et al. [51] reported that oleanolic acid administration attenuated A*β*-induced memory loss in a rat model of AD by maintaining synaptic plasticity of the hippocampus. There are also several studies reporting the remedial effects of OS chemical components, such as ursolic acid [52, 53], apigenin [54, 55], and luteolin [56–58], on cognitive deficits in various animal models. Thus, these components are most plausibly able to explain the effects of OS extract in the OBX animals. However, the present study does not exclude the possibility that luteolin-7-O-glucuronide and apigenin-7-O-glucuronide also contributed to OS extract's antidementia effects. Indeed, chemical evidence demonstrates that luteolin-7-O-glucuronide or apigenin-7-O-glucuronide can be deconjugated by the activity of *β*-glucuronidase expressed in macrophages, thereby resulting in the active form of aglycones: luteolin and apigenin [59, 60].

Moreover, the comparison based on a molecular docking analysis [61] showed a considerable structure similarity between luteolin-7-O-glucuronide and DNP bound to human acetylcholinesterase, suggesting the potential of luteolin-7-O-glucuronide to act like DNP in the brain. Therefore, OS extract's antidementia action is likely due to these triterpenoid and flavonoid components included in OS extract. Nevertheless, because the antidementia effects of OS implicated multiple mechanisms including hippocampal neurogenesis, stimulation/protection of central cholinergic systems, and restoration of VEGF expression, further studies are required to clarify the potential contribution of each component on the antidementia action of OS.

The present results revealed that OS, which has long been used as Ayurvedic medicine and tea herbs/a cuisine material in Vietnam and other Southeast Asian countries, possessed antidementia effects and overall underlying mechanisms similar to those of DNP. Considering that it takes more than decades before cognitive dysfunction of patients is diagnosed as AD, our findings offer a rationale for long-term use of OS aiming at prevention rather than at AD therapy and distinctive features of this extract as compared to DNP, which is used only to improve cognitive dysfunctions of AD patients.

## 5. Conclusion

This study demonstrates that the administration of OS attenuates cognitive deficits and neurohistological damages caused by OBX. The actions of OS are at least in part mediated by enhancement of central cholinergic systems and VEGF expression.

## Figures and Tables

**Table 1:** Primer sets for target genes in real-time PCR.

Gene		Primer sequence
VEGF	Forward	5′-AGGAGAGATGAGCTTCCTACAG-3′
	Reverse	5′-TCACCGCCTTGGCTTGTCACAT-3′

VEGFR2	Forward	5′-GGGATGGTCCTTGACTACAG-3′
	Reverse	5′-ACTGGTAGCCACTGGTCTGG-3′

*β*-Actin	Forward	5′-CATCCGTAAAGACCTCTATGCCAAC-3′
	Reverse	5′-ATGGAGCCACCGATCC ACA-3′

## Data Availability

The data used to support the findings of this study are included within the article and in its Supplementary Materials.

## Supplementary Materials

Supplementary Materials Quantities of chemical constituents in ethanol extract of *Ocimum sanctum* using high-performance liquid chromatography analysis.
