# Supplementary material for: Ocimum sanctum Linn. Extract Improves Cognitive Deficits in Olfactory Bulbectomized Mice via the Enhancement of Central Cholinergic Systems and VEGF Expression
Source: Evid Based Complement Alternat Med. 2021 Jun 30;2021:6627648. doi: 10.1155/2021/6627648 (PMC8266455; doi:10.1155/2021/6627648)
Supplement: Supplementary Materials — Quantities of chemical constituents in ethanol extract of Ocimum sanctum using high-performance liquid chromatography analysis. [file 6627648.f1.docx]

**High-performance liquid chromatography (HPLC) analysis**

**Materials and Methods**

HPLC separation and quantification of some compounds in OS extract was performed at 25°C using a 5-µm Vertisep® C18 column (250 mm × 4.6 mm; Vertical Chromatography Co., Ltd, Bangkok, Thailand). The mobile phases, A (water with 0.01% TFA acid) and B (methanol), were employed for the analysis of flavonoids (luteolin, apigenin, luteolin-7-*O*-glucuronide, and apigenin-7-*O*-glucuronide). The elution programs were as follows: from 0 to 30 min with isocratic 30% (v/v) B, from 30 to 35 min with linear gradient 30 – 80% (v/v) B, and from 35 to 45 min with isocratic 30% (v/v) B. The flow rate was 1.5 ml/min, and the injection volume was 20 µl. These analytes were monitored by a UV- VIS detector at 330 nm. For the analysis of triterpenoid components, a binary eluent of 95% acetonitrile (A) and 5% water (B) was used as a mobile phase and applied under isocratic conditions at a flow rate of 0.4 ml/min. Compound elution was monitored by a UV- VIS detector at 210 nm.

a) Preparation of standard solutions and calibration

Ursolic acid, apigenin and luteolin were obtained from Chengdu Biopurify Phytochemicals (China); oleanolic acid from Sigma- Aldrich (USA); apigenin-7-*O*-glucuronide from Chengdu Herbpurify (China). Luteolin-7-*O*-glucuronide was isolated from *Ocimum sanctum* by Dr. Tai Van Nguyen (Department of Phytochemistry, NIMM, Vietnam, data not shown). The compounds were weighed and dissolved in methanol. The final volume was adjusted to 10 ml. The stock solution was diluted to obtain different concentrations. Triplicate analyses were carried out for each concentration. The calibration curve was obtained by calculating peak areas against concentration.

b) Preparation of samples

The OS extract was weighed and sonicated with methanol for 20 minutes. The final volume was adjusted to 10 ml. Each sample was filtered through a 0.45μm cellulose acetate membrane (Whatman®, Merck, Darmstadt, Germany), and 20 µL of each sample was directly injected into HPLC (Shimadzu LC-20A system, Tokyo, Japan).

c) Method validation

The HPLC validation procedure was processed according to analytical standards. Limit of detection (LOD) and limit of quantification (LOQ) were determined for the precision of the method. LOD and LOQ were established at a signal to noise ratio (S/N) of three and ten, respectively. LODs and LOQs were experimentally verified by six replicate injections.

**Result**

The quantities of ursolic acid, oleanolic acid, apigenin, luteolin, luteolin-7-*O*-glucuronide, and apigenin-7-*O*-glucuronide, in the crude OS extract, were determined using precision HPLC analysis [RSD=0.027-1.264% (<2%), Supplemental table]. Based on this analysis, the OS extract contained: 1.02 % ursolic acid, 1.48% oleanolic acid, 0.10% apigenin, 0.11% luteolin, 3.06% luteolin-7-*O*-glucuronide, and 0.85% apigenin-7-*O*-glucuronide. Chromatograms of flavonoids (apigenin, luteolin, luteolin-7-*O*-glucuronide, and apigenin-7-*O*-glucuronide), triterpenoids (ursolic acid and oleanolic acid), and the crude OS extract are shown in supplemental figure S1 and 2.

**Supplemental Table.** Analytical validation parameters results of HPLC analysis method.

| Compound | Calibration equation | r^2^ | LOD µg/mL | LOQ µg/mL | RSD (%) |
| --- | --- | --- | --- | --- | --- |
| Apigenin | y = 66772x - 122714 | 0.9999 | 0.054 | 0.178 | 0.082 |
| Luteolin | y = 51380x - 2181 | 0.9998 | 0.037 | 0.122 | 0.027 |
| Apigenin-7-*O*-glucuronide | y = 42914x - 65219 | 0.9999 | 0.118 | 0.354 | 1.264 |
| Luteolin-7-*O*-glucuronide | y = 20351x - 301495 | 0.9994 | 0.328 | 0.984 | 1.032 |
| Ursolic acid | y = 24072x + 27687 | 0.9999 | 0.151 | 0.503 | 0.686 |
| Oleanolic acid | y = 28365x - 25571 | 0.9999 | 0.539 | 1.797 | 0.437 |

RSD: Relative Standard Deviation = (Standard Deviation/Average) X 100


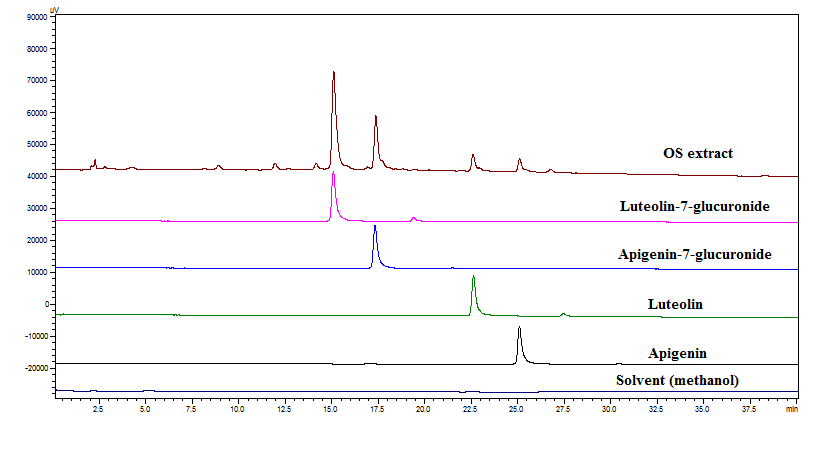


Supplemental Fig S1. HPLC chromatograms of luteolin, apigenin, luteolin-7-*O*-glucuronide and apigenin-7-*O*-glucuronide in OS extract


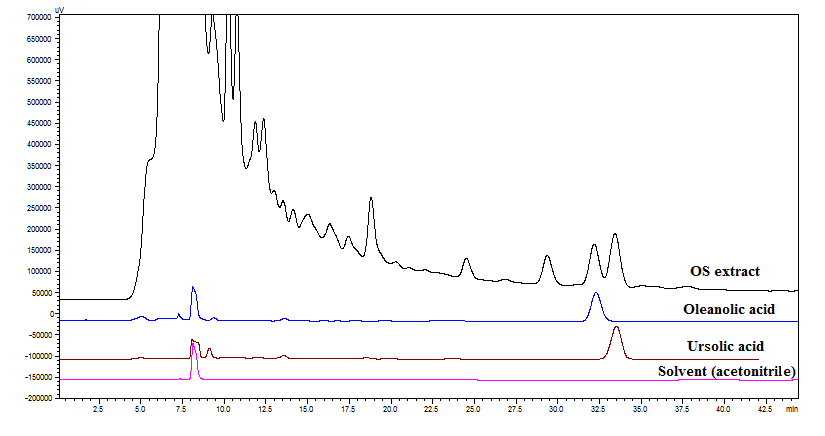


Supplemental Fig S2. HPLC chromatograms of ursolic acid and oleanolic acid in OS extract
